# Development and characterization of 10 microsatellite markers in the Cape horseshoe bat, *Rhinolophus capensis* (Chiroptera, Rhinolophidae) and cross-amplification in southern African *Rhinolophus* species

**DOI:** 10.1186/s13104-015-1465-5

**Published:** 2015-09-26

**Authors:** Nicolas Nesi, David S. Jacobs, Kevin Feldheim, Jacqueline M. Bishop

**Affiliations:** Department of Biological Sciences, University of Cape Town, 7701 Cape Town, South Africa; Pritzker Laboratory for Molecular Systematics and Evolution, Field Museum of Natural History, 1400 South Lake Shore Drive, Chicago, IL 60605 USA

**Keywords:** *Rhinolophus capensis*, Microsatellites, Cross-amplification, Genetic connectivity, Rhinolophidae

## Abstract

**Background:**

The Cape horseshoe bat, *Rhinolophus capensis*, is endemic to the Cape region of South Africa. Coalescent analysis of mitochondrial DNA sequence data suggests extensive historical gene flow between populations despite strong geographic variation of their echolocation call phenotype. Nevertheless the fine-scale genetic structure and evolutionary ecology of *R. capensis* remains poorly understood. Here we describe the development of 10 novel polymorphic microsatellite loci to investigate of the dispersal ecology of *R. capensis* and to facilitate taxonomic studies of *Rhinolophus* species in southern Africa.

**Findings:**

We report 10 microsatellite primer pairs that consistently amplify scorable and polymorphic loci across 12 African rhinolophid species. Initial analysis of two populations of *R. capensis* from South Africa revealed moderate to high levels of allelic variation with 4–14 alleles per locus and observed heterozygosities of 0.450–0.900. No evidence of linkage disequilibrium was observed and eight of the loci showed no departure from Hardy–Weinberg equilibrium. Cross-species utility of these markers revealed consistently amplifiable polymorphic loci in eleven additional rhinolophid species.

**Conclusions:**

The cross-amplification success of the microsatellites developed here provides a cost-effective set of population genetic marker for the study of rhinolophid evolutionary ecology and conservation in southern Africa.

**Electronic supplementary material:**

The online version of this article (doi:10.1186/s13104-015-1465-5) contains supplementary material, which is available to authorized users.

## Findings

The Cape horseshoe bat (*Rhinolophus capensis*) has a predominantly coastal distribution encompassing several major biomes in South Africa [1]. Despite evidence for extensive historical gene flow the species is characterised by geographically structured variation in echolocation frequency which appears to be closely coupled to local environmental conditions [2]. Such phenotypic variation is evident in a number of rhinolophids (1; DSJ, unpublished data). Here we develop and test a suite of microsatellite markers (1) to test hypotheses of adaptive evolution in *R. capensis* and (2) to better understand population genetic structuring and the recent evolutionary history of African rhinolophids.

Twenty-six species of horseshoe bats have been described in sub-Saharan Africa with 11 occurring in the southern African countries of Botswana, Lesotho, Mozambique, Namibia, South Africa, Swaziland, and Zimbabwe [1]. The taxonomy and true number of species of African *Rhinolophus* nevertheless remains unresolved because of the highly convergent morphology observed across taxa. Currently species delimitation is based on slight variations in morphological measurements and echolocation call frequency [1, 3] but there is much ambiguity in these characters as a result of extensive intraspecific phenotypic variation and the true diversity of species is likely to be underestimated when using these methods alone. The recent inclusion of genetic data has uncovered a number of species complexes in the African rhinolophids (e.g. *R. hildebrandtii* and *R. darlingi*) [4, 5]. The use of an integrative approach combining molecular techniques and morphological analyses has enabled the recognition and description of several new species and will undoubtedly contribute to the improved taxonomic resolution of African rhinolophid diversity.

Microsatellite loci are characterised by high polymorphism and co-dominance which make them ideal genetic markers for use in population genetic studies. These molecular markers have also been widely used to study demographic and ecological processes within species and to resolve taxonomic problems among recently diverged lineages [6]. Their value in such studies is further enhanced by the fact that microsatellite markers primarily developed for a specific taxon can also be used on closely related species, making them a cost effective tool for taxonomic, population and conservation genetic studies.

In this study microsatellite markers were isolated following an enrichment protocol developed by Glenn and Schable [7]. To construct the library we used genomic DNA (gDNA) from both *R. capensis* and a closely related taxa Dent’s horseshoe bat, *R. denti.* Total DNA was extracted using a DNeasy Blood and Tissue Kit (Qiagen) from biopsy wing punch samples (3 mm) collected from the wing or tail membrane. To increase the amount of enriched fragments, a ‘recovery’ PCR was performed after the initial round of enrichment. Reactions were performed in a final volume of 25 μL containing 1X PCR buffer (10 mM Tris–HCl, 50 mM KCl, pH 8.3), 1.5 mM MgCl_2_, 0.16 mM of each dNTP, 10 × BSA, 0.5 μM of the Super SNX24 forward primer, 1U *Taq* DNA polymerase, and approximately 25 ng enriched gDNA fragments. Thermal cycling, performed in an MJ Research DYAD, was performed as follows: 95 °C for 2 min followed by 25 cycles of 95 °C for 20 s, 60 °C for 20 s, and 72 °C for 90 s, and a final elongation step of 72 °C for 30 min. Subsequent PCR fragments were cloned using the TOPO-TA Cloning kit (Invitrogen) following the manufacturer’s protocol. DNA sequencing was performed using the BigDye® Terminator v3.1 Cycle Sequencing Kit (Applied Biosystems) and run on an ABI 3730 DNA Analyzer. From the resulting libraries we identified 50 *R. capensis* and 55 *R. denti* clones that contained repetitive elements with sufficient flanking regions for primer development. A total of 55 primer pairs were designed from the positive clones both libraries using the web-based program Primer3Plus [8].

For the initial PCR trials, amplification of 21 and 15 microsatellite loci from the *R. capensis* and *R. denti* libraries, respectively, were tested using eight individuals of each species. PCR was carried out in a final volume of 20 µL containing ~25 ng of DNA template, 0.5 µM of forward and reverse primers, 1X PCR buffer, 1.5 mM MgCl_2_, 0.2 mM of each dNTP and 5 U/µL Kapa Taq DNA polymerase (Kapa Biosystems). PCR amplification was performed under the following conditions: initial denaturation at 95 °C for 5 min, 35 cycles of 95 °C for 45 s, annealing for 45 s at 48–60 °C (gradient tests) and 72 °C for 45 s, followed by a final extension of 72 °C for 10 min. To confirm amplification and identify unambiguous loci PCR products were first visualised on a 3 % agarose gel under UV. Of the initial 36 loci tested, seven loci isolated from *R. capensis* and three loci from *R. denti* consistently amplified scorable loci in our target study species. These loci were then used to determine levels of polymorphism using a sample of 40 *R. capensis* individuals. All loci were then tested for cross-species amplification and polymorphism in 11 additional rhinolophids distributed in southern and central Africa using 2–5 individuals from across their respective ranges (geographic localities indicated in the Additional file 1). The 5′ end of each forward primer was labelled with a fluorescent dye (6-FAM, NED, PET and VIC) for visualisation of the PCR products. For the initial characterisation of loci amplification for each locus was performed individually using 40 *R. capensis* individuals from two populations (see Table 1 for PCR conditions). PCR products were then pooled for each individual in two different multiplex panels (Table 1).

**Table 1:** Characterisation and PCR conditions for 10 microsatellite loci in the Cape horseshoe bat, *Rhinolophus capensis*

Locus	Repeat motif	Primer sequences (5′–3′)	Dye	Temp. annealing (°C)	Panel	Expected product size (bp)	Observed size range (bp)	Population	*n*	N_A_	N_E_	H_O_	H_E_	P_HW_	PIC	GenBank accession number
RCA1	(ATAG)_10_	F: TGG GAA ATC TCC AAA CCT TCC	VIC	55	1	560	554–582	Table farm	19	8	4.57	0.737	0.781	0.092	0.789	KP842268
		R: GGG CTG CCT CAA CAT AAT ACC						Heidehof	20	6	4.87	0.700	0.795	0.288		
RCA2	(AG)_32_	F: GTG AAC CTA CTT GCT TGA CTC ACC	6-FAM	54	1	519	492–538	Table farm	14	12	6.87	0.786	0.855	0.030	0.903	KP842262
		R: TGG CCA GTT GAA GAC AGT CTC C						Heidehof	17	14	9.63	0.529	0.896	0.0001*		
RCA3	(TAGA)_14_	F: AGC AGC CTC CAT AAT AAC ATA AGC	VIC	50	2	420	398–418	Table farm	20	5	3.37	0.700	0.704	0.756	0.708	KP842266
		R: AAC TCT TAT CCT AAT CTC ACC TCC						Heidehof	20	6	4.08	0.750	0.755	0.657		
RCA4	(TG)_20_	F: GTA TGA GTC CTT TGT CAG ATA GAG G	6-FAM	52	2	375	374–384	Table farm	19	6	3.48	0.632	0.713	0.008	0.685	KP842263
		R: TGG TAA CAA TGC AGT GTA ATG G						Heidehof	20	5	3.68	0.850	0.729	0.667		
RCA5	(TC)_26_	F: GCT CAT GCA TGG TAG GCA ATG G	VIC	57	1	311	288–312	Table farm	18	4	2.48	0.833	0.597	0.121	0.631	KP842265
		R: AGA CAT TCA GGT CTC CAC ACT CC						Heidehof	18	7	3.17	0.667	0.685	0.506		
RCA6	(GATA)_12_	F: GCA GAG CAG AAT GTC AAC ATC C	PET	52	2	258	238–262	Table farm	19	4	3.04	0.632	0.672	0.122	0.634	KP842264
		R: AGT AGC CAT CCC TTT CAA TCC						Heidehof	20	5	3.21	0.850	0.689	0.572		
RCA7	(ATAG)_10_	F: GCC TTC ATA GAG CTG ATA TGT TGG	NED	56	1	234	228–244	Table farm	20	5	3.90	0.900	0.744	0.359	0.690	KP842267
		R: GAG CCT GCC ATT CTA CAA TCC						Heidehof	20	4	3.49	0.800	0.714	0.893		
RDE1	(TCTA)_11_	F: TGT TAC ATC TTG GTC CCT TGA GG	NED	53	1	400	394–410	Table farm	20	5	3.82	0.650	0.739	0.710	0.685	KP842271
		R: GCT GCA TAT GGG CTG AGA TAA AC						Heidehof	20	4	3.37	0.850	0.704	0.326		
RDE2	(AC)_25_	F: TCA CTC ACT TCT CTC GAC TCC	PET	53	1	400	387–409	Table farm	18	7	3.10	0.500	0.677	0.106	0.642	KP842269
		R: AGA CCA CAA CTC TTA ACT CTG C						Heidehof	20	7	2.87	0.450	0.653	0.006		
RDE3	(TC)_24_	F: CCT TCT CAG TTA CTA TTA CCT CAC C	NED	53	2	336	332–388	Table farm	19	12	9.13	0.737	0.891	0.059	0.872	KP842270
		R: TGC TCT AAC TCC TCA CAC ACC						Heidehof	20	11	5.51	0.750	0.840	0.514		

Number of individuals genotyped (*n*), number of alleles observed per locus (N_A_), effective number of alleles (N_E_), observed (H_O_), and expected heterozygosity (H_E_), Probability of deviation from Hardy–Weinberg Equilibrium (P_HW_), Polymorphic information content (PIC)

* P values significant after Bonferroni correction at α = 0.05 (p < 0.0025)

For cross-species testing of the loci PCR amplification was carried out using a QIAGEN Multiplex PCR Kit on two multiplex panels containing primers labelled with different fluorescent dyes (seven and two loci); one locus (RCA3, T 50 °C) was amplified alone using the protocol described earlier. Multiplex PCR amplifications were performed in a final volume of 20 µL containing 10 µL of QIAGEN Multiplex PCR Master Mix, 2 µL of the 10× primer mix with each primer at a final concentration of 2 µM and 1 µL of DNA template. Thermal cycling, performed in an Applied Biosystems Veriti 96-Well Thermal Cycler, was performed as follows: 95 °C for 15 min; 35 cycles of 94 °C for 30 s, 57 °C for 90 s, 72 °C for 60 s and a final step of 60 °C for 30 min. PCR products separated by capillary electrophoresis on an AB3730 DNA analyser (Central Analytical Facilities, Stellenbosch, South Africa). Alleles were sized using an internal size standard (GeneScan600LIZ) and scored using the software GeneMarker 2.6.3 (SoftGenetics).

To characterise variation at these loci individuals of *R. capensis* were genotyped from two colonies representing the full geographic extent of the species (Table Farm: −33.28, 26.42; Heidehof: −34.62, 19.50). For each locus a number of parameters were calculated in GenAlEx 6.5 [9], including the number of alleles observed (N_A_), effective number of alleles (N_E_), observed and expected heterozygosities (H_O_ and H_E_). Tests of Hardy–Weinberg equilibrium (P_HW_) and tests of linkage of disequilibrium (LD) between loci and populations were calculated in Genepop 4.3 [10]. MicroChecker 2.2.3 [11] was used to detect typing errors and null alleles. The polymorphism information content (PIC) for each locus was assessed with Cervus 3.0.7 [12].

Across the ten loci the number of alleles per locus, N_A,_ ranged from 4 to 14, while the effective number of alleles per locus, N_E,_ ranged from 2.48 to 9.63 in *R. capensis* (Table 1). The observed (H_O_) and expected (H_E_) heterozygosities ranged from 0.450 to 0.900 and 0.597 to 0.896, respectively. Deviation from Hardy–Weinberg equilibrium (HWE) was not found evident except for locus RCA2 (*P* < 0.05 after Bonferroni adjustment). Null alleles was found for two loci (Table Farm, RDE2 r = 0.122; Heidehof, RDE2, r = 0.172, RCA2, r = 0.201). All of the loci revealed moderate to high PIC values (0.631–0.904).

Cross-amplification tests of the 10 loci were successful on all the 11 species selected, and 98 % of loci were polymorphic (Table 2). The number of *Rhinolophus* species that showed polymorphism across the 10 loci ranged from 11 to 12 and the number of polymorphic loci within taxa ranged from 9 to 10.

**Table 2:** Cross amplification results in 11 additional *Rhinolophus* species from southern Africa

Taxa	Collection country	*n*	Loci tested									
			RCA1	RCA2	RCA3	RCA4	RCA5	RCA6	RCA7	RDE1	RDE2	RDE3
*Rhinolophus blasii*	Malawi, RSA, Zambia, Zimbabwe	5	2/5	5/5	5/5	6/5	3/5	7/5	4/5	6/5	6/5	6/5
			500–546	488–500	402–454	364–388	200–304	254–270	214–236	376–418	379–401	298–344
*Rhinolophus clivosus*	RSA, Mozambique	5	5/5	6/5	4/5	6/5	8/5	5/5	5/5	5/5	5/5	8/5
			534–566	484–524	406–422	366–376	272–324	270–286	236–256	424–454	373–387	330–398
*Rhinolophus damarensis*	Namibia, RSA	5	4/5	7/5	5/5	6/5	7/5	6/5	4/5	4/5	6/5	7/5
			528–548	494–534	406–422	376–392	268–332	262–278	232–244	398–490	379–397	298–342
*Rhinolophus darlingi*	RSA, Zimbabwe	5	1/3	3/5	6/5	7/5	4/5	8/5	6/5	7/5	4/5	7/5
			500	478–500	418–442	364–392	200–256	244–296	212–248	374–454	395–401	302–338
*Rhinolophus denti*	Botswana, Namibia, RSA	5	4/4	4/4	4/4	2/4	6/4	3/4	3/5	4/4	5/4	4/3
			512–540	510–530	406–422	370–376	258–340	242–258	228–248	386–402	391–401	312–350
*Rhinolophus fumigatus*	Namibia, Zimbabwe	5	5/5	5/5	6/5	4/5	8/5	6/5	5/5	7/5	5/5	4/5
			536–560	478–528	414–442	368–394	228–292	302–330	220–236	438–472	374–402	302–308
*Rhinolophus hildebrandtii*	RSA, Zimbabwe	5	3/5	2/5	3/5	4/5	8/5	6/5	5/5	4/5	3/5	2/5
			544–552	474–480	414–432	370–376	248–284	258–296	226–240	380–398	385–391	308–312
*Rhinolophus landeri*	Zimbabwe	2	4/2	2/2	2/2	2/2	2/2	4/2	4/2	1/2	2/2	4/2
			536–554	464–486	424–436	362–376	238–254	266–282	224–238	376	379–391	272–306
*Rhinolophus mossambicus*	Zambia, Zimbabwe	4	4/3	6/3	6/3	4/4	5/3	4/4	5/3	6/3	4/3	2/3
			544–556	478–520	398–430	360–366	224–242	278–292	224–240	402–446	361–391	300–304
*Rhinolophus simulator*	Botswana, RSA, Zambia, Zimbabwe	5	5/5	8/5	6/5	4/5	4/5	3/5	5/5	5/5	7/5	7/5
			542–574	488–528	398–430	370–384	294–304	250–258	228–256	386–402	381–409	318–352
*Rhinolophus swinnyi*	RSA	5	3/5	6/5	4/5	1/5	3/5	3/5	2/5	4/5	3/5	5/5
			532–540	500–522	402–418	364	268–314	246–254	232–236	386–398	381–393	332–346

For each species on the top row: the number of alleles/genotypes scored. On the bottom row: allele size ranges

GPS locality for each individual are indicated in the Additional file 1

The 10 microsatellite markers reported here will be highly useful for a wide range of population and evolutionary genetic studies in the African Rhinolophidae. Microsatellite markers presented here, together with mitochondrial and additional nuclear markers, will provide valuable tools to resolve the systematic and taxonomic relationships of several species complexes of *Rhinolophus*.

## Availability of the supporting data

The microsatellite sequences are available through the National Centre for Biotechnology Information (http://www.ncbi.nlm.nih.gov); GenBank accession numbers KP842262–KP842271.

## Additional file

10.1186/s13104-015-1465-5 Geographic information of the individuals used for the cross-amplification tests.

## References

[1] Monadjem A, Taylor PJ, Cotterill FPDW, Schoeman MC (2010). Bats of Southern and Central Africa a biogeographic and taxonomic synthesis.

[2] Odendaal LJ, Jacobs DS, Bishop JM (2014). Sensory trait variation in an echolocating bat suggests roles for both selection and plasticity. BMC Evol Biol.

[3] Csorba G, Ujhelyi P, Thomas N. Horseshoe bats of the world (Chiroptera: Rhinolophidae). Alana Books; 2003. p. 160i.

[4] Taylor PJ, Stoffberg S, Monadjem A, Schoeman MC, Bayliss J, Cotterill FPD (2012). Four new bat species (*Rhinolophus hildebrandtii* Complex) reflect Plio-Pleistocene divergence of dwarfs and giants across an afromontane archipelago. PLoS One.

[5] Jacobs DS, Babiker H, Bastian A, Kearney T, van Eeden R, Bishop JM (2013). Phenotypic convergence in genetically distinct lineages of a *Rhinolophus* species complex (mammalia, chiroptera). PLoS One.

[6] Selkoe KA, Toonen RJ (2006). Microsatellites for ecologists: a practical guide to using and evaluating microsatellite markers. Ecol Lett.

[7] Glenn TC, Schable NA (2005). Isolating microsatellite DNA loci. Methods Enzymol.

[8] Untergasser A, Nijveen H, Rao X, Bisseling T, Geurts R, Leunissen JAM. Primer3Plus, an enhanced web interface to Primer3. Nucleic Acids Res. 2007; 35 (Web Server issue):W71–4. http://www.bioinformatics.nl/cgi-bin/primer3plus/primer3plus.cgi/. 10.1093/nar/gkm306 PMC1933133 17485472

[9] Peakall R, Smouse PE (2012). GenALEx 6.5: genetic analysis in Excel. Population genetic software for teaching and research-an update. Bioinformatics.

[10] Rousset F (2008). GENEPOP’007: a complete re-implementation of the GENEPOP software for Windows and Linux. Mol Ecol Resour.

[11] Van Oosterhout C, Hutchinson WF, Wills DPM, Shipley P (2004). Micro-Checker: software for identifying and correcting genotyping errors in microsatellite data. Mol Ecol Notes.

[12] Kalinowski ST, Taper ML, Marshall TC (2007). Revising how the computer program CERVUS accommodates genotyping error increases success in paternity assignment. Mol Ecol.

