# Supplementary material for: Development and characterization of 10 microsatellite markers in the Cape horseshoe bat, Rhinolophus capensis (Chiroptera, Rhinolophidae) and cross-amplification in southern African Rhinolophus species
Source: BMC Res Notes. 2015 Sep 26;8:477. doi: 10.1186/s13104-015-1465-5 (PMC4584015; doi:10.1186/s13104-015-1465-5)
Supplement: Supplementary file 1 — 10.1186/s13104-015-1465-5 Geographic information of the individuals used for the cross-amplification tests. [file 13104_2015_1465_MOESM1_ESM.docx]

**Additional file 1** Geographic information of the individuals used for the cross-amplification tests.

|  |  |  |  |  |  |  |  |
| --- | --- | --- | --- | --- | --- | --- | --- |
| **Species** | **Code specimens** | **ID number** | **Site** | **Country** | **Name Locality** | **Latitude** | **Longitude** |
| *blasii* | Rbl1 | 3006MtMRbls01 | MTM | Malawi | Mont Mulanje | S16°1’39.81’’ | E35°32’33.80’’ |
| *blasii* | Rbl2 | GKC240911Rbl01 | GKC | South Africa | Gatkop cave | S24°37’09.9” | E27°39’11.1” |
| *blasii* | Rbl3 | 19062012Rda02CHK | CHK | Zimbabwe | Chikuti | S17°07’00” | E29°54’00” |
| *blasii* | Rbl4 | 150613GMRbl09 | GM | Zimbabwe | Grand-Manica | S32°19’0.01” | E18°58’59.98” |
| *blasii* | Rbl5 | KAL7291011Rbl02 | KAL | Zambia | Kalenda | S14°43’54.72” | E26°54’01.62” |
| *clivosus* | Rcl1 | 160613MMRcl01 | MM | Mozambique | Monaci | S32°43’33.56” | E18°53’2.12” |
| *clivosus* | Rcl2 | 22412Rcl01PC | PC | South Africa | Peppercorn cave | S24°08’46.3” | E29°10’10.3” |
| *clivosus* | Rcl3 | 071110Rcl02BM | BM | South Africa | Bendigo mine | S34°3’57.2” | E23°13’20.2” |
| *clivosus* | Rcl4 | 29032011Rcl04DHL | DHL | South Africa | De Hel | S33°04´.59” | E19°04´.58” |
| *clivosus* | Rcl5 | 301010Rcl05GHS | GHS | South Africa | Geelhoutsbos | S33°36’10.44” | E24°12’0.36” |
| *damarensis* | Rda1 | 13042010Rda01DAN | DAN | Namibia | Dante’s cave | S19°23’49.2” | E17°53’06.0” |
| *damarensis* | Rda2 | 7410R?3ARN | ARN | Namibia | Arnhem cave | S22°42’7.2” | E18°5’49.2” |
| *damarensis* | Rda3 | 16112009R?20OR | OR | South Africa | Vioolsdrif, Orange river | S28°42’07.6” | E17°32’21.1” |
| *damarensis* | Rda4 | 21711Rda7SF | SF | South Africa | Soetfontein | S28°23’05.5” | E23°03’02.9” |
| *damarensis* | Rda5 | 14012010R?22UN | UN | South Africa | Uintjiesberg Farm, Carnarvon | S30°49’31.8” | E22°32’11.04” |
| *darlingi* | Rdr1 | 08062012R?02MTP | MTP | Zimbabwe | Matobo | S20°30’19.92” | E28°26’29.53” |
| *darlingi* | Rdr2 | 15042011Rda01EH | EH | South Africa | Elandshoek mine | S25°22’33.1” | E30°41’33.2” |
| *darlingi* | Rdr3 | 13042011Rda01SUD | SUD | South Africa | Sudwala cave | S25°22’32.7” | E30°41’32.1” |
| *darlingi* | Rdr4 | 19062012Rda02CHK | CHK | Zimbabwe | Chikuti | S17°07’00” | E29°54’00” |
| *darlingi* | Rdr5 | 15042011Rda03EH | EH | South Africa | Elandshoek mine | S25°22’33.1” | E30°41’33.2” |
| *denti* | Rde1 | Rde1/121111RdeGWC04 | GW | Botswana | Gcwihaba cave | S20°01’15” | E21°21’19.2” |
| *denti* | Rde2 | Rde2/180711RdeKGB05 | KGB | South Africa | Koegelbeen | S28°39’11.3” | E23°20’55.2” |
| *denti* | Rde3 | Rde3/210711RdeSF01 | SF | South Africa | Soetfontein | S28°23’05.5” | E23°03’02.9” |
| *denti* | Rde4 | Rde4/131111rdeWH03 | WH | Botswana | Waxhu | S19°46’38.58” | E21°02’31.08” |
| *denti* | Rde5 | Rde5/140410Rde01GHB | GHB | Namibia | Ghaub cave | S19°29’2.4” | E17°46’40.8” |
| *fumigatus* | Rfu1 | 13410DANRfu01 | DAN | Namibia | Dante’s cave | S19°23’49.2” | E17°53’06.0” |
| *fumigatus* | Rfu2 | 12410Rfu3LUD | LUD | Namibia | Ludwig cave | S19°24’02.62” | E16°59’23.0” |
| *fumigatus* | Rfu3 | 19612Rfu01CHK | CHK | Zimbabwe | Chikuti | S17°07’00” | E29°54’00” |
| *fumigatus* | Rfu4 | 230613Rfu01JET | JET | Zimbabwe | Jiri Estate Triangle | S31°22’45.91’’ | E20°56’20.86’’ |
| *fumigatus* | Rfu5 | 19613MWBRfu11 | MWB | Zimbabwe | Muuyuweburi | S32°40’47.02’’ | E19°46’58.54’’ |
| *hildebrandtii* | Rhi1 | 190613MWBRhi02 | MWB | Zimbabwe | Muuyuweburi | S32°40’47.02’’ | E19°46’58.54’’ |
| *hildebrandtii* | Rhi2 | 270613KPRhi01 | KP | Zimbabwe | Kapatamukombe | S16°23’43.47’’ | E30°51’26.74’’ |
| *hildebrandtii* | Rhi3 | 090613BTRhi01 | BT | South Africa | Big Baobab Tree | S22°30´2.16” | E30°37´59.16” |
| *hildebrandtii* | Rhi4 | 7612Rhi02MTP | MTP | Zimbabwe | Matobo | S20°30’19.92” | E28°26’29.53” |
| *hildebrandtii* | Rhi5 | 10612Rhi07MUS | MUS | Zimbabwe | Mushandike | S20°7’25.11” | E30°35’56.49” |
| *landeri* | Rla1 | 220612MPCRsw01 | MPC | Zimbabwe | Mana pools Chitake | S16°5’28.07” | E29°27’51.04” |
| *landeri* | Rla2 | 220612MPCRsw02 | MPC | Zimbabwe | Mana pools Chitake | S16°5’28.07” | E29°27’51.04” |
| *mossambicus* | Rmo1 | 31102011Rhi01LH | LH | Zambia | Leopard’s Hill | S15°36’07.32” | E28°43’27.78” |
| *mossambicus* | Rmo2 | 281011Rhi01KAL2 | KAL | Zambia | Kalenda | S14°43’54.72” | E26°54’01.62” |
| *mossambicus* | Rmo3 | 24102011RhiCC01 | CC | Zimbabwe | Chinhoyi cave | S17°21’24.84” | E30°07’45.3” |
| *mossambicus* | Rmo4 | 24102011RhiCC02 | CC | Zimbabwe | Chinhoyi cave | S17°21’24.84” | E30°07’45.3” |
| *simulator* | Rsi1 | 281011Rsi01KAL7 | KAL7 | Zambia | Kalenda 7 | S14°44’15.78” | E26°54’18.42” |
| *simulator* | Rsi2 | 15411Rsi02EH | EL | South Africa | Elandshoek mine | S25°22’33.1” | E30°41’33.2” |
| *simulator* | Rsi3 | 18102011Rsi01LOB | LOB | Botswana | Lobatse | S25°14’11.1” | E25°39’41.04” |
| *simulator* | Rsi4 | 240911Rsi03KGC | KGB | South Africa | Koegelbeen | S28°39’11.3” | E23°20’55.2” |
| *simulator* | Rsi5 | 26612Rsi07MAB | MAB | Zimbabwe | Mabura cave | S17°53’48.30” | E29°22’0.11” |
| *swinnyi* | Rsw1 | 040207Rsw1KWT | KWT | South Africa | King Williams Town, Pirie Forest | S32°43´00.34” | E27°17´00.00” |
| *swinnyi* | Rsw2 | 040207Rsw2KWT | KWT | South Africa | King Williams Town, Pirie Forest | S32°43´00.34” | E27°17´00.00” |
| *swinnyi* | Rsw3 | DSJKSM16 | SM | South Africa | Kokstad Mine | S30°31’00.00” | E29°29’00.00” |
| *swinnyi* | Rsw4 | DSJSPF11 | SPF | South Africa | Steenkampskraal | S30°58´40.0” | E18°37´51.0” |
| *swinnyi* | Rsw5 | DSJSPF1 | SPF | South Africa | Steenkampskraal | S30°58´40.0” | E18°37´51.0” |
|  |  |  |  |  |  |  |  |
|  |  |  |  |  |  |  |  |
